# Nanoslot metasurface design and characterization for enhanced organic light-emitting diodes

**DOI:** 10.1038/s41598-021-88641-6

**Published:** 2021-04-29

**Authors:** Kyungnam Kang, Seongmin Im, Changhun Lee, Jungho Kim, Donghyun Kim

**Affiliations:** 1 School of Electrical and Electronic Engineering, Yonsei University, Seoul, 03722 South Korea; 2 Department of Information Display, Kyung Hee University, Seoul, 02447 South Korea

**Keywords:** Nanoscience and technology, Optics and photonics

## Abstract

We investigate bottom-emitting organic light-emitting diodes (B-OLEDs) integrated with metasurface (MS) to analyze the effect of the structural parameters on the output performance. The performance of the MS-integrated B-OLED (MIB-OLED) is evaluated by out-coupling efficiency (OCE) and reflection of the ambient light, while attention is paid mainly to dielectric capping and metal structure of MS that may influence excitation of surface plasmon (SP). The results suggest that layer thicknesses affect the performance by as much as 10% for the OCE and up to 32% for reflectance. The OCE is in general weakly affected by the structural parameters of MS. In contrast, the reflectance characteristics are found to be dominated by localized SP that is largely determined by the length and the width of a unit slot of MS. An optimization factor introduced to evaluate the performance based on out-coupling power to the radiation mode and reflectance of MIB-OLEDs confirms that integration with MS improves performance by 16% over conventional planar structure. In particular, MIB-OLED is found to enhance OCE by 51% with Lambertian-like pattern. Enhanced performance is experimentally confirmed. The findings provide insights on how to optimize the MS structure to produce MIB-OLEDs with enhanced out-coupled power and contrast ratio.

## Introduction

Organic light-emitting diodes (OLEDs) now account for a significant market share of the display industry in high-end applications such as foldable and flexible smartphones, TVs, and other display devices. This is because OLEDs exhibit many advantages, e.g., light weight, wide color gamut, good flexibility, short response time, and true black state^1–5^. However, there exists a trade-off between light extraction enhancement and retainment of low reflectance of ambient light: in more detail, since OLEDs are composed of multiple films sandwiched by the top and the bottom metallic mirrors with thickness on the order of nanometers, the light-emitting power can be enhanced by the micro-cavity effect^6–8^. On the other hand, strong reflection from inner metallic electrodes leads to a very low contrast ratio (CR) in OLEDs under bright ambient light condition. CR may be expressed as

1 $$\text{CR}=\frac{{L}_{on}+{R}_{D}{L}_{amb}}{{L}_{off}+{R}_{D}{L}_{amb}}$$

where *L*_*on*_ and *L*_*off*_ denote luminance of on- and off-state pixels of OLEDs, respectively, whereas *L*_*amb*_ is the ambient luminance^9^. *R*_*D*_ is the luminous reflectance of OLEDs, given by

2 $${R}_{D}=\frac{{\int }_{{\lambda }_{1}}^{{\lambda }_{2}}V(\lambda )R(\lambda )S(\lambda )d\lambda }{{\int }_{{\lambda }_{1}}^{{\lambda }_{2}}V(\lambda )S(\lambda )d\lambda }$$

where *V(λ)* represents the eye sensitivity spectrum. *R(λ)* is the reflectance at the OLED surface and *S(λ)* an optical spectrum of ambient light. Equation (1) implies that reduced *R*_*D*_ would increase contrast. Reflectance *R*_*D*_ of external light may also affect contrast of an image. Therefore, both CR and *R*_*D*_ are important in OLEDs. In order to diminish the reflection of the ambient light, a circular polarizer composed of a quarter-wavelength plate and a linear polarizer has been often adopted in OLEDs^10,11^. Although a circular polarizer can keep the reflection of ambient light under 4–6%, it may increase panel thickness thus reduce flexibility and inevitable power absorption losses bring about more than 50% cutback of the out-coupling efficiency (OCE)^9^, which represents the efficiency of extracting photons out of the cavity and is expressed as a ratio of external quantum efficiency (EQE) to internal quantum efficiency (IQE), i.e., OCE = EQE/IQE.

For this reason, many researchers have investigated polarizer-free high-contrast OLED structures to replace circular polarizers with, for instance, absorbing transport layers^9,12,13^, low reflectance material as an electrode^14–16^, black cathodes^17–20^, anti-reflection coating^21–24^, neutral-density attenuation filters^25^, destructive interference structures^26^, and tandem structures^27,28^. Despite various approaches that have been reported, however, there are still issues which stem primarily from trade-offs between low reflectance of ambient light and the OCE into the radiation mode (air mode). Also, the additional layers in the electrically active region often cause an adverse effect on the electrical properties and lifetime of OLEDs. In addition, previous works have been largely limited to multiple planar film structures.

Recently, metasurface (MS), which consists of periodic arrays of subwavelength structure and thereby attains extraordinary photonic properties, has been employed for tuning plasmonic resonances from infrared to visible regime in applications that include biochemical sensing^29–32^, photovoltaic solar cells^33^, optical imaging^34–36^, Raman spectroscopy^37^, and color filtering^38–41^. In particular, nearly perfect absorbers in the visible range composed of MS with metallic elements have been developed using colloidally synthesized silver nanocubes^42^ and sub-wavelength silver hole arrays^43^. Because most designs of MS is based on strong frequency-dependent resonance, spectral characteristics tend to be narrow-banded. Therefore, proposals have been made to broaden the bandwidth with near-perfect absorption in the visible regime by taking nanocomposite (Au/SiO_2_)^44^, phase-change material^45^ and metallic mixed-slot arrays^46^. MS has been also used to enhance light extraction and modulate the emission pattern of OLEDs^47,48^. Despite such great potential to absorb light in the visible range, MS has not been adopted in OLEDs for the replacement of circular polarizers and the effects of geometrical parameters of MS have not been fully evaluated for OLEDs.

In this paper, therefore, we investigate MS-integrated bottom-emitting OLEDs (MIB-OLEDs) to explore the effects of various geometrical parameters on the performance measures such as OCE and the luminous reflectance. The MS is designed to consist of sub-wavelength mixed-slot arrays of gold combined with a cathode from a bottom-emitting OLED (B-OLED). We focus on thicknesses of layers, such as metal of MS and dielectric layer, which amplify surface plasmon polariton (SPP). We also consider excitation of localized surface plasmon (LSP) by varying structural parameters of a unit slot. Initially, we address the validity of calculation using finite element method (FEM) by comparing with the classical electromagnetic model, then we analyze the OCE and the luminous reflectance of MIB-OLEDs with respect to the layer thicknesses and other parameters such as length and width of the unit slot. After all, an optimized structure of MS is proposed for enhanced out-coupling power to the radiation mode and the CR of an OLED.

## Numerical model and method

### 3D model for MIB-OLED

**Figure 1:**
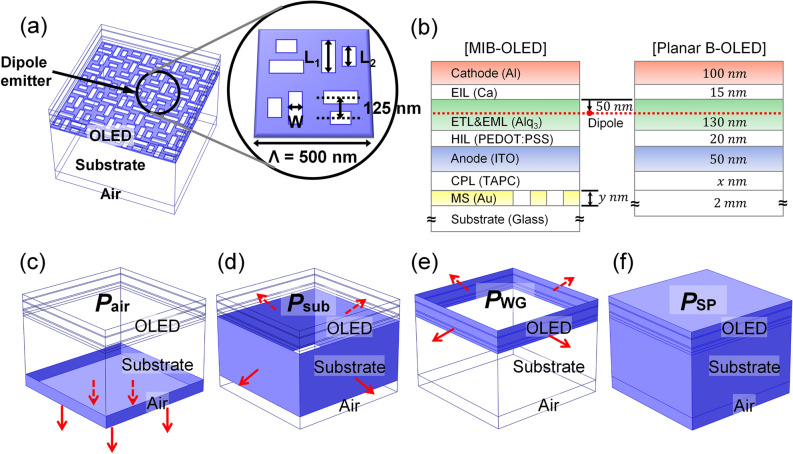
(**a**) 3D schematic illustration of the calculation model with MS integrated B-OLED (MIB-OLED, MS layer in blue). A dipole emitter is located at the center of the device. Emitted light from the dipole emitter is directed to the bottom through glass substrate to air ambience. One of the unit cells of the MIB-OLED is shown enlarged in the inset. Lengths and widths of the eight cut-out slots are indicated to *L*_1_, *L*_2_, and *W* (*Λ* = 500 nm). (**b**) 2D layer cross-section of the MIB-OLED vs. conventional planar B-OLED with materials and thicknesses of the model. The vertical location of the dipole emitter is 50 nm apart from EIL for constructive two-beam interference of micro-cavity effects. (**c**–**f**) Schematic illustration of the surface and the domain used to calculate the coupling mode power of the B-OLED. The amplitude of Poynting vectors through blue-colored surfaces of (**c**) air, (**d**) substrate, and (**e**) OLED device represents the power coupled to the air, substrate, and WG modes. The red arrows represent the direction of Poynting vector in each mode. (**f**) Plasmon loss is calculated by the integration of power dissipation in the whole volume of the OLED structure.

We use a simple 3D model for a MIB-OLED as illustrated in blue in Fig. 1a. The MS is based on conventional visible range perfect absorber and consists of a periodic array of unit cells, which is shown enlarged in the inset: with multiple slots for the MS, the design may take advantage of many design parameters to optimize LSP absorption spectrum. The MS to absorb ambient light was assumed on a glass substrate, above which transparent anode of indium tin oxide (ITO) and a thin dielectric layer, called a capping layer (CPL), are stacked. The CPL is used as an optical functional layer to optimize the OCE or tune the spectral distributions^49^. The unit MS cell is a gold layer with eight cut-out slots filled with a dielectric material (*n* = 1.7), which is the same as the material of the CPL. Two cut-out slots with an identical width of *W* and different lengths of *L*_*1*_ and *L*_*2*_ are combined together in quadrant sections rotated orthogonally. The period of the unit MS is fixed at *Λ* = 500 nm. For comparison of the optical performances, we have calculated wavelength-dependent spectra and OCE.

The layer cross-section of a MIB-OLED is illustrated in Fig. 1b, which also shows the cross-section of a conventional planar B-OLED structure. The planar B-OLED uses 100-nm aluminum as a top cathode, 15-nm calcium as an electron injecting layer (EIL), 130-nm tris-(8-hydroxyquinoline) aluminum (Alq_3_) as both the electron-transport layer (ETL) and emission layer (EML), 20-nm poly(3,4)-ethylendioxythiophene doped with poly(styrene sulfonate) (PEDOT:PSS) as a hole injection layer (HIL), 50-nm thick ITO as a bottom anode, 1,1-bis[(di-4-tolylamino)phenyl]cyclohexane (TAPC) as a dielectric CPL (thickness: *x* nm) on a thick glass substrate, as shown in Fig. 1b. The refractive indices of the materials used in the B-OLED model were obtained by ellipsometry or taken from the literature^50,51^. For the OCE, simulation was performed at the wavelength of 520 nm which coincides with the photoluminescence peak of Alq_3_^52^. A dipole emitter is isotropic and 50 nm apart from the Ca layer (EIL) having a *δ*-distributed emission zone based on two-beam interference of micro-cavity effects for the radiation mode. The thicknesses of ETL and EML were determined to optimize the OCE in the vicinity of *λ* = 520 nm so as to maximize multiple-beam interference due to micro-cavity effects. For the MIB-OLED model, a mixed-slot layer was added below CPL (height: *y* nm). The coupling-power to radiation, substrate, and waveguide (WG) modes were calculated by surface integration of amplitudes of Poynting vectors in the domain relevant to each mode, as shown in Fig. 1c–e. The plasmon mode and absorption losses were calculated by volume integration of power dissipation in the whole OLED device, as shown in Fig. 1f.

The MIB-OLED model is designed to consider the interaction between the top cathode (aluminum) and the bottom gold MS for leading to improved absorption. The top cathode layer plays the role of an optical mirror to reflect incident light, while the bottom MS acts as a scatterer causing excitation of surface plasmon (SP) modes. Two crucial mechanisms that may be involved in resonant transmission are known to be the excitation of SPP and LSP^53,54^. The formation of SPP modes is attributed to the interference of multiple waves scattered by slot arrays and depends on the arrangement and thickness of organic and dielectric layers. LSP resonance (LSPR) is also affected by the shape and the size of the unit slot because LSP modes are generated at the edges of the slot.

The optical properties of MIB-OLEDs were analyzed based on the OCE and the luminous reflectance. IQE of fluorescent OLEDs limited to 25% due to the use of singlet excitons in the cavity affects the OCE little. To increase the CR of OLEDs, the reflectance from the ambient light should be diminished. On the other hand, the emitted light from dipole emitters needs to be efficiently out-coupled to the radiation mode to achieve high power efficiency. To achieve an optimum with this consideration, we set two groups of geometrical parameters for the MS. The first group of parameters concerns the thickness of CPL (*d*_*CPL*_) and the gold MS layer (*d*_*MS*_) that are associated primarily with the resonance between the top metal and the bottom gold layer. The other group is the one consisting of the length of the shorter slot (*L*_*2*_) and the slot width (*W*) in connection with the effect of localized fields.

### Numerical method

For the 3D calculation in the far-field, finite element method (FEM) was used under appropriate boundary conditions^55–57^. FEM has been used to calculate electromagnetic properties of an optical structure in many studies for the broad applicability and robustness in modeling and is often preferred over other techniques such as finite difference time domain (FDTD) and discontinuous galerkin time-domain (DGTD) method because it employs flexible mesh structure with reduced time load. We have used a commercial software COMSOL Multiphysics with its wave optics module in which Maxwell equations are numerically solved in small meshes that divide calculation domains. In the calculation of the OCE, a scattering boundary condition was used at all the outer boundaries of the 3D OLED device to eliminate reflection at the outer boundary. Although a pixel of OLEDs has a size on the order of tens of micrometers, a square pixel was assumed here to be 2 μm × 2 μm in the lateral plane in order to minimize workstation memory usage. The light emission from exciton in the EML is modeled as the radiation of a Hertzian point dipole^58^.

Because plasmonic absorption may increase temperature of OLEDs thus decrease the device lifetime, we have also analyzed thermal property of MIB-OLED using wave-coupled heat transfer equation^59^. The power dissipation calculated in the optical model was replaced as the heat source term in the heat transfer equation. Wave-coupled time-dependent heat transfer equation was then solved by FEM to obtain the temperature distribution in the OLED model. Amplitude of the dipole moment was matched to the emitted normal power intensity at 30 mW/cm^2^ with B-OLEDs^60^. For thermal analysis, we used properties of organic materials reported in the past^61–63^. With external ambient temperature set to be *T*_*amb*_ = 293.15 K (room temperature), the local temperature of MIB-OLED was found to rise up to *T*_*max*_ = 293.18 K, i.e., the rise of temperature poses an insignificant effect so as to change the lifetime of OLEDs.

In the calculation of reflectance, periodic structure was modeled with periodic boundary conditions at the outermost side boundaries. Port boundary conditions were used at the lowermost boundary of the ambience and at the uppermost of the cathode to model plane waves from an ambient region with normal incidence to the device. Because thick glass substrate acts as an incoherent layer, coherent calculation results were averaged using the equispaced thickness method (ETM) with three additional thicknesses of the glass substrate:

3 $${d}_{1}=0, \;\; {d}_{2}=\frac{\lambda }{{6n}_{g}}, \;\; {d}_{3}=\frac{2\lambda }{{6n}_{g}},$$

where *λ* is the wavelength of the incident ambient light and *n*_*g*_ is the refractive index of the glass substrate. The averaged electric field intensity considering incoherency was obtained by

4 $${\left|E\left(x,y,z\right)\right|}^{2}=\frac{1}{3}\sum_{j=1}^{3}{\left|E\left(x,y,z,{d}_{j}\right)\right|}^{2},$$

where *E(x, y, z, d*_*j*_*)* represents the electric field obtained by the coherent calculation at the *x*, *y*, and *z* position of the B-OLED structure in the Cartesian coordinates with the *j*-th thickness of glass substrate using FEM (*j* = 1, 2, 3). Using the calculated electric field intensities, the spatial distribution of light absorption can be obtained. The total reflectance of the B-OLED considering incoherency of the glass substrate can then be easily calculated by *R*_*tot*_ = 1 *−* *A*, where *A* represents the absorbance of the whole device, because the top metal cathode suppresses light transmission through the cathode. The accuracy of the ETM was demonstrated in previous studies^64,65^. The reflectance of thin-film organic solar cells calculated by the ETM with three additional incoherent layers shows that the deviation from the exact solution can be maintained under 2%^64^. Although averaging over a larger number of incoherent layers may enhance the accuracy of the results, we used three incoherent layers to reduce the calculation time. To calculate the luminous reflectance *R*_*D*_, D65 (a standard light source) was chosen to be the ambient light source. The accuracy of FEM is affected by the mesh size, i.e., with a reduced mesh size, accuracy is improved at the expense of long calculation time and more computational resource. We set the maximum mesh size to be 60 nm to optimize calculation time and accuracy.

## Results and discussion

### Effect of layer thicknesses

First of all, power flow and mode analysis of B-OLED have been performed to validate the reliability of the FEM calculation. The calculated power flows coincide with the general optical characteristics of OLEDs. Mode analysis of B-OLED also shows overall in excellent agreement between FEM calculation and point dipole model in the classical electromagnetic theory. For investigation of MIB-OLEDs, two groups of geometrical parameters of MS were varied: *d*_*CPL*_ and *d*_*MS*_ related to SPP and *L*_*2*_ and *W* to the excitation of LSP. In this section, we explore the effect of *d*_*CPL*_ and *d*_*MS*_ on the OCE and reflectance.

#### Out-coupling efficiency

**Figure 2:**
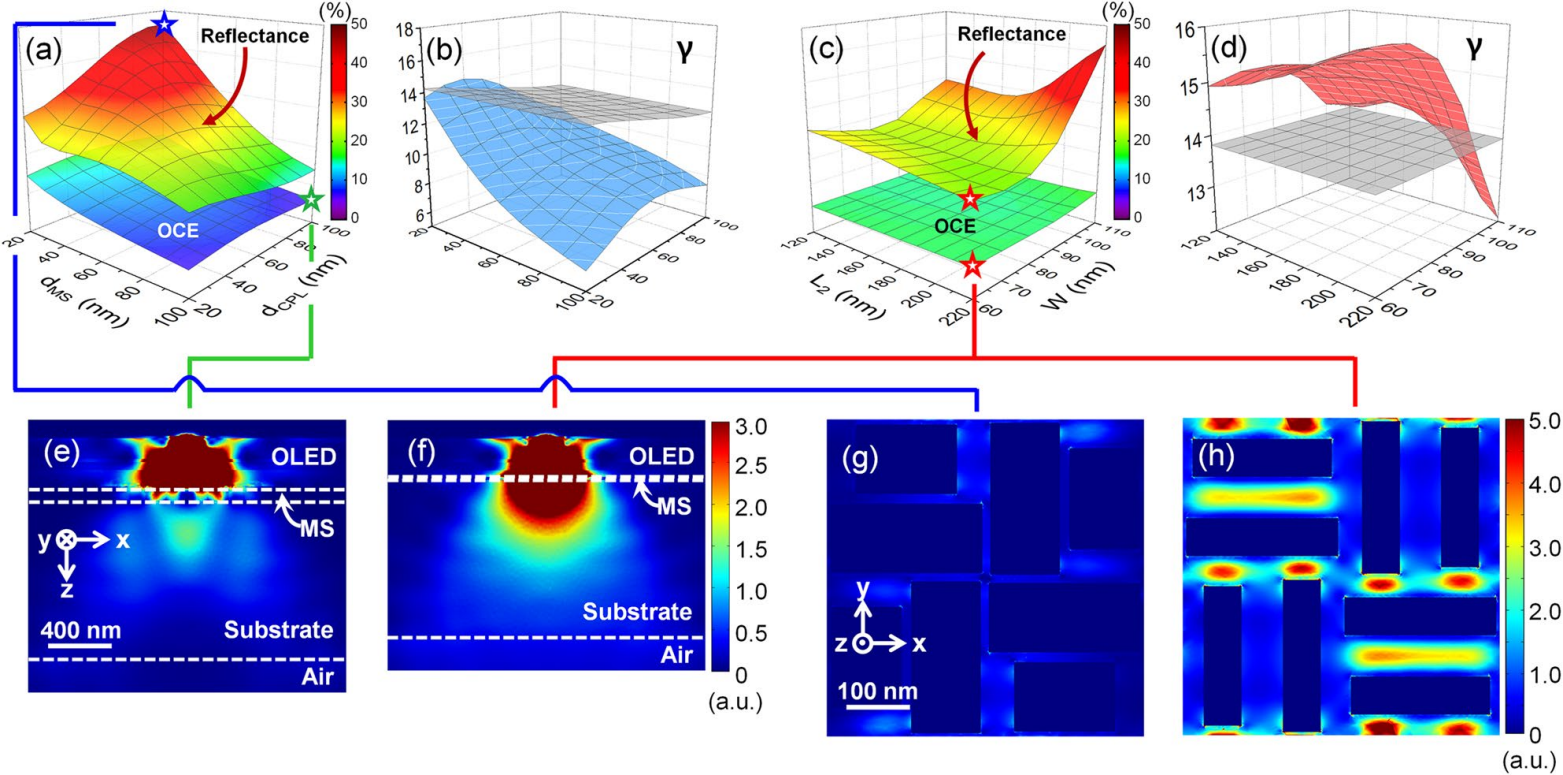
(**a**) OCE and the luminous reflectance while the thickness of CPL and MS is varied in the range of 20–100 nm. *L*_*1*_, *L*_*2*_, and *W* are fixed to 240, 160, and 110 nm, respectively. (**b**) Optimization factor (*γ*) of MIB-OLED (in blue) and conventional B-OLED (in gray) with respect to the thickness of CPL and MS in the range of 20–100 nm. (**c**) OCE and the luminous reflectance with respect to *L*_*2*_ and *W. L*_*2*_ and *W* are varied, respectively, in the range of 120–220 nm and 60–110 nm. (**d**) Optimization factor (*γ*) of the MIB-OLED (in red) and the conventional B-OLED (in gray). *d*_*CPL*_, *d*_*MS*_, and *L*_*1*_ are fixed at 40, 20, and 240 nm. Due to the lack of MS in conventional B-OLED, *γ* remains as constant for conventional B-OLED along the *d*_*MS*_ axis. Power flow of MIB-OLEDs: (**e**) spatial distribution in the *zx* plane for MS with *L*_*1*_ = 240 nm, *L*_*2*_ = 160 nm, *W* = 110 nm, *d*_*MS*_ = 100 nm, and *d*_*CPL*_ = 100 nm assuming horizontal dipole emission at *y* = 0. The parameter set is in (**a**) marked with a green star symbol. (**f**) For optimum MS with *L*_*1*_ = 240 nm, *L*_*2*_ = 220 nm, *W* = 60 nm, *d*_*MS*_ = 20 nm, and *d*_*CPL*_ = 40 nm, as marked in (**c**) with a red star symbol. (**g**) Spatial distribution in the *xy* plane by external light incidence (*λ* = 550 nm) for MS with *L*_*1*_ = 240 nm, *L*_*2*_ = 160 nm, *W* = 110 nm, *d*_*MS*_ = 20 nm, and *d*_*CPL*_ = 100 nm, as marked in (**c**) with a blue star symbol, and (**h**) for the same optimum MS marked with a red star symbol in (**c**).

*d*_*CPL*_ and *d*_*MS*_ were varied to understand the resonance effect of the meta-structure on the out-coupling mechanism. The out-coupling ratios of MIB-OLEDs with respect to *d*_*CPL*_ and *d*_*MS*_ are presented in Fig. 2a. The MS composed of two slots has different lengths of *L*_*1*_ = 240 nm and *L*_*2*_ = 160 nm. The width of a unit slot *W* is set to 110 nm. *d*_*CPL*_ and *d*_*MS*_ were varied from 20 to 100 nm in a step of 10 nm. The CPL is assumed to be a non-absorbing dielectric layer sandwiched between top and bottom metal layers.

Change in *d*_*CPL*_ may cause multiple resonances inside the OLED cavity due to the redistribution of photonic density of states in the quantum mechanical approach^53, 66^. Because multiple interferences are governed by the thickness of the dielectric layer between top and bottom metal, an increased thickness leads to the fluctuation in the OCE. In Fig. 2a, *d*_*CPL*_ increases the OCE up to *d*_*CPL*_ = 50 nm, after which the OCE is reduced regardless of *d*_*MS*_ even as the CPL becomes thicker. This is because *d*_*CPL*_ = 50 nm satisfies the condition of constructive interference. Interestingly, even though the highest out-coupling power ratio from the whole emission power is obtained at *d*_*CPL*_ = 50 nm, higher power flow to the air mode can be achieved with *d*_*CPL*_ = 60 nm. The parameter set that leads to an optimum for the OCE and the power flow into the air mode can be disparate, which is why the whole coupling power to the multiple modes is varied by the way that a dipole emitter is configured, e.g., under resonant cavity condition.

On the other hand, the OCE is decreased as *d*_*MS*_ increases. High extinction of gold makes MS actively absorb emitted light from the emission layer in thick MS. When *d*_*MS*_ increases from 20 to 100 nm, therefore, the OCE falls dramatically below half. Here, we have required *d*_*MS*_ ≥ 20 nm because light absorption of ambience through MS is essential to reduce background noise. In the case of *d*_*CPL*_ = 60 nm and *d*_*MS*_ = 20 nm, the OCE reaches a maximum at 15.9%. In contrast, the minimum OCE was obtained as 6.0% with *d*_*CPL*_ = 100 nm and *d*_*MS*_ = 100 nm.

#### Reflection characteristics

To enhance the CR of OLEDs, reflection caused by the ambient light at the bottom glass surface should be minimized. The mixed slots tend to act as a perfect absorber of light ambience in the visible wavelength range. Like the case of the OCE, two different lengths of the unit slot *L*_*1*_ and *L*_*2*_ are fixed at *L*_*1*_ = 240 nm and *L*_*2*_ = 160 nm. The slot width *W* is selected to be 110 nm. The reflectance of light ambience, when the light is incident along the normal direction toward the glass substrate, was calculated varying *d*_*CPL*_ and *d*_*MS*_. The range was set equally to the out-coupling calculation between 20 and 100 nm in a step of 10 nm. Calculation of reflectance was performed in the wavelength range from λ_1_ = 400 to λ_2_ = 700 nm and converted to the luminous reflectance *R*_*D*_ using Eq. (2).

Figure 2a shows the luminous reflectance varying *d*_*CPL*_ and *d*_*MS*_. Most notably, thicker MS reduces reflectance regardless of *d*_*CPL*_. Obviously, a thick MS layer of gold increases absorption of light emitted from a dipole emitter and of external light ambience. The reflectance is also reduced when SPP waves are excited. Because the reflectance characteristics are associated with the interaction between top cathode and bottom MS layers, the thickness of MS may affect the dielectric layer thickness conditioned for efficient SPP excitation, e.g., with *d*_*MS*_ = 20 nm, the luminous reflectance (*R*_*D*_) becomes the lowest at *R*_*D*_ = 29.0% at *d*_*CPL*_ = 20 nm. When *d*_*MS*_ > 60 nm, *d*_*CPL*_ corresponding to the minimum *R*_*D*_ is switched from 20 to 90 or 100 nm. Note that *R*_*D*_ is dominated by the characteristics in the wavelength range of 500–600 nm, in which thicker MS changes the dielectric layer thickness to maximize absorption caused by the SP mode, related to spectra of eye sensitivity and ambient light. Overall, the minimum reflectance was obtained as 13.9% at *d*_*CPL*_ = 90 nm and *d*_*MS*_ = 100 nm. The maximum luminous reflectance was found to reach up to 46.2% with a difference due to the layer thickness measured to be 32.3%.

#### Optimization of the overall characteristics

In order to find an optimized set of *d*_*CPL*_ and *d*_*MS*_ based on quantitative evaluation, we have defined an optimization factor (*γ*) as

5 $$\gamma \, = \, P_{rad} (1 - R_{D} ),$$

where *P*_*rad*_ and *R*_*D*_ represent the out-coupled power to the radiation mode and the luminous reflectance. (1 – *R*_*D*_) works as the CR. The definition of *γ* reflects the desired qualities of OLEDs for high out-coupled optical power into the radiation mode and low reflectance of the ambient light and is presented in Fig. 2b.

Figure 2b shows optimization factors of MIB-OLED and conventional B-OLED in meshed surface. *γ* of the B-OLED does not depend on *d*_*MS*_ (no MS layer) and is affected weakly by *d*_*CPL*_. Note that a circular polarizer adopted to diminish reflectance of ambient light is generally known for good performance of reducing the reflectance down to *R*_*D*_ = 5%. However, light emission from a dipole emitter is also significantly reduced by half after a circular polarizer, i.e., the overall out-coupled power to the radiation mode falls to 50% and the luminous reflectance appears only as *R*_*D*_ = 5% in the conventional B-OLED. With a B-OLED, *γ* is obtained as *γ* = 14.23 at *d*_*CPL*_ = 20 nm. As the CPL is thicker, the optimization factor is decreased with a slope of *Δγ/Δd*_*CPL*_ = − 0.023 nm^−1^, i.e., for an increase of 10 nm in *d*_*CPL*_, *γ* is reduced by 1.6% with respect to the peak value. The optimization factor is finally obtained as 12.39 when *d*_*CPL*_ = 100 nm. Because the layer thicknesses of a B-OLED are designed to maximize the out-coupling power at *λ* = 520 nm, a thicker CPL causes a change of constructive interference in the OLED cavity, thereby reduces the out-coupled power to the radiation mode. In contrast, *γ* of the MIB-OLED in Fig. 2b (blue surface) was shown to excel that of B-OLED, i.e., *γ*_*max*_ = 14.35 when *d*_*CPL*_ = 40 nm and *d*_*MS*_ = 20 nm. Although this by itself represents enhancement by merely 3.9% over conventional B-OLEDs, further improvement can be made by optimizing MS structures.

### Effect of structural parameters of MS

To achieve further enhancement of OCE and CR, we investigate the effect of structural parameters. The length of a shorter slot *L*_*2*_ and slot width *W* in the MS were varied, which influence SP localization. In this analysis, we have set *d*_*CPL*_ = 40 nm and *d*_*MS*_ = 20 nm.

#### Out-coupling efficiency and reflection characteristics

For simplicity, *L*_*1*_ is fixed at 240 nm. *L*_*2*_ and *W* were changed in the range of *L*_*2*_ = 120–220 nm and *W* = 60–110 nm. In Fig. 2c, OCE reaches the minimum when *W* = 90 nm. The OCE of the B-OLED tends to slightly increase with longer *L*_*2*_ and larger *W*. This is conceptually obvious because longer *L*_*2*_ and larger *W* are directly connected with a smaller area of the absorbing metal and thus higher likelihood of light waves to transmit through MS. However, LSP has a weak effect on the emitted light from the dipole emitter, therefore the OCE depends only weakly on *L*_*2*_ and *W*. On the other hand, structural parameters have a more visible effect on the reflectance of the ambient light. With *L*_*2*_ = 210 nm, the minimum reflectance was obtained as 26.1% in the case of *W* = 70 nm. As *L*_*2*_ becomes longer, *W* corresponding to the minimum reflectance decreases, i.e., LSP is well excited from the appropriate balance of the slot structure. In the case of long *L*_*2*_ and large *W*, the reflectance dramatically increases up to 46.4% at *L*_*2*_ = 220 nm and *W* = 110 nm. The large area of the slot structure tends to suppress excitation of LSP and causes reflectance of the ambient light to be high.

#### Optimization of the overall characteristics

The optimization factor *γ* between B-OLED and MIB-OLED is described in Fig. 2d. The flat surface in gray presents the optimization factor of the B-OLED at *d*_*CPL*_ = 40 nm. The optimization factor of a B-OLED was obtained as 13.81 regardless of the parameters with no mixed-slot structure. In contrast, the optimization factor of a MIB-OLED is much higher than that of B-OLED for the majority of the combinations of *L*_*2*_ and *W*, mostly for those with *L*_*2*_ < 190 nm and *W* < 100 nm. The maximum was achieved as *γ*_*max*_ = 16.02 when *L*_*2*_ = 220 nm and *W* = 60 nm, i.e. the enhancement associated with MS reaches 16% (= 16.02/13.81), compared to the conventional B-OLED. These structural parameters produce an OCE of 15.6% and reflectance at 26.9% and the OCE shows enhancement by 51% (= 15.6/10.3).

The enhancement achieved by optimization of MS can be better understood from spatial distribution of power flow shown in Fig. 2e–h. The distribution in the cross-section (*zx* plane) shows that sub-optimal thick MS in Fig. 2e [green star symbol in Fig. 2a] absorbs light emission and decreases OCE to 6.03%. In contrast, Figure 2f presents the power flow with optimal MS corresponding to the case of red star symbol in Fig. 2c, in which light emitted from a dipole is efficiently coupled to MS without significant absorption and thus improves the OCE. The power distribution in the horizontal (*xy*) plane for external light incidence at *λ* = 550 nm is shown in Fig. 2g,h. In Fig. 2g with sub-optimal MS which corresponds to blue star symbol in Fig. 2a, we observe extremely weak light localization, which causes coupling of light to the cavity to be difficult and thereby reflectance to increase. On the other hand, we can clearly see efficient localization of light using optimum MS in Fig. 2h [red star symbol in Fig. 2c], which tends to reduce reflectance. Enhanced emission of light in OLED connected with efficient plasmonic localization is in good agreement with experimental data^67,68^.

One may wonder about the performance on flexible substrates such as polyimide (*n* = 1.75). When the MIB-OLED corresponding to red star symbol in Fig. 2c has the substrate replaced with polyimide, OCE was improved to 17.8%. This is because an increase of substrate index reduces index mismatch between substrate and organic layers. This causes light to be trapped less in organic layers under total internal reflection and results in higher OCE. On the other hand, reflectance increases slightly to 28.5% on polyimide substrate. Flexible substrates may also have finite surface curvature, which indices more diffusive emission with larger etendue. Because SPP excitation and LSP absorption spectrum on a curved surface tend to broaden at the expense of poorer contrast^69^, it may be presumed that OCE would improve with an increase of reflectance on flexible substrates for a range of surface curvature.

While the enhancement in itself represents significant performance improvement of a MIB-OLED, we emphasize that the optimization was not performed in the full parameter space to save computation resource and shorten the calculation time. Therefore, we expect further enhancement to be made with more efficient optimization algorithms based, for example, on machine learning techniques to scan the complete parameters spaces.

#### Angular distribution characteristics

Angular emission distribution of MIB-OLED is calculated comparing to one of conventional planar B-OLED. We choose the MIB-OLED which has optimized structural parameters corresponding to the case of red star symbol in Fig. 2c. Angular emission distribution is obtained by combining means of FEM which calculates power flow on the top surface of glass substrate with analytical method which considers the multiple reflection within glass substrate and angle conversion from glass substrate to ambient region. Reliability of combined FEM with analytical method is validated by the calculated result of angular emission distribution of planar B-OLED using generalized Fabry-Pérot model^52^.

**Figure 3:**
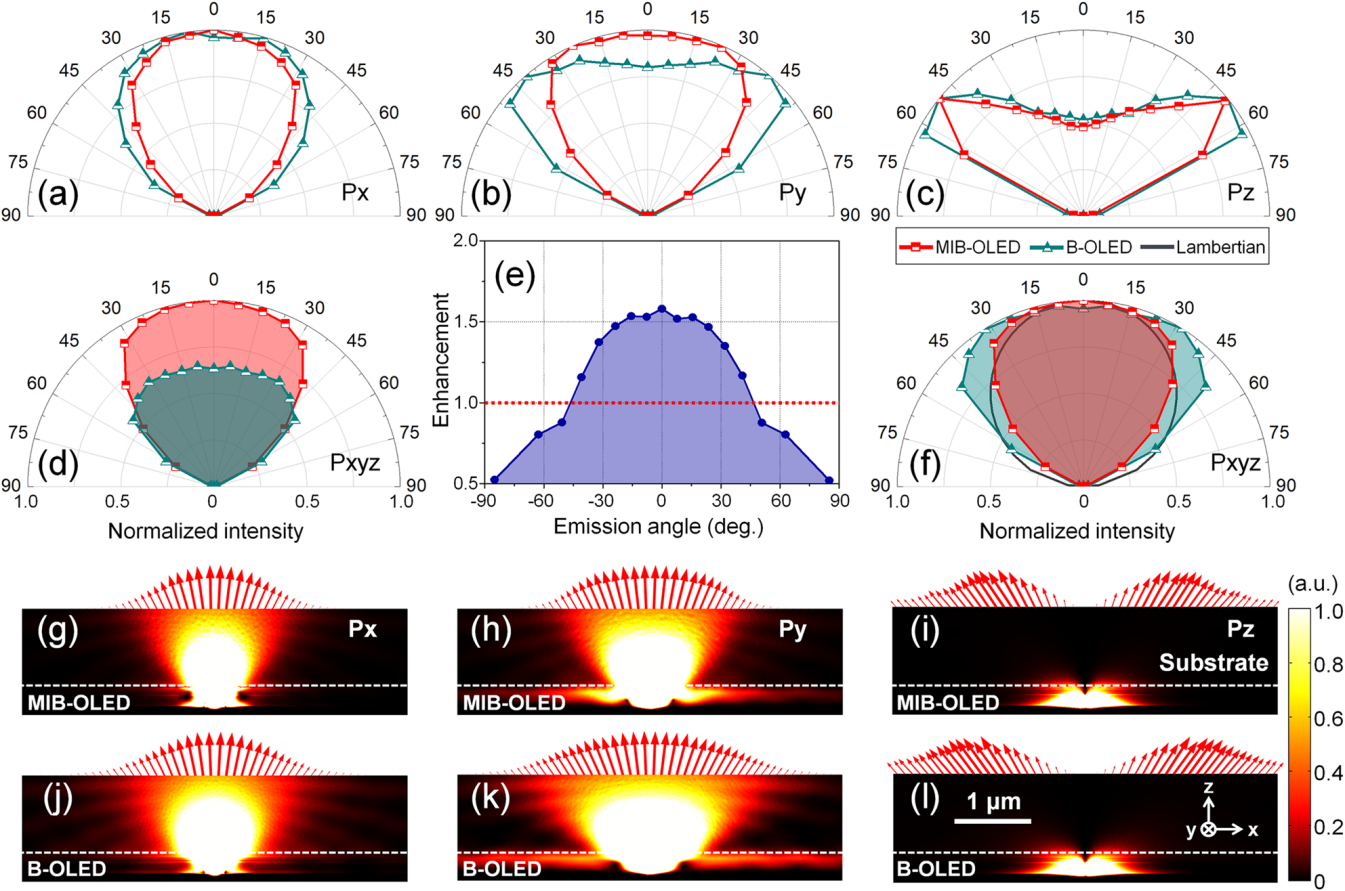
(**a**) Normalized angular emission characteristics of MIB-OLED and planar B-OLED with dipole emitters horizontally aligned to (**a**) *x*-axis (*Px*) and (**b**) *y*-axis (*Py*), and that are vertically aligned along the (**c**) *z*-axis (*Pz*). The coordinate orientation is given in Fig. 2e,g. (**d**) Angular emission characteristics of MIB-OLED and planar B-OLED normalized by the maximum value of MIB-OLED with isotropic dipole orientation (*Pxyz*). (**e**) Enhancement of out-coupled intensity of MIB-OLED in reference to that of planar B-OLED with respect to the emission angle. Dotted line in red indicates where out-coupled intensity of MIB-OLED and planar B-OLED are equal. (**f**) Normalized angular emission characteristics of MIB-OLED and planar B-OLED with isotropic dipole orientation in comparison with a Lambertian pattern. Spatial distribution of time-averaged power with respect to the dipole emitter orientation: (**g**–**i**) MIB-OLED and (**j**–**l**) planar B-OLED in the *zx* plane. Red arrows indicate spatial distribution of time-averaged Poynting vectors on the top surface of a glass substrate.

Figures 3a–c show the normalized angular emission distribution of MIB-OLED (red line with square symbol) and planar B-OLED (cyan line with triangle symbol) with different dipole orientations along the horizontal *x* and *y*-axis, and vertical axis of *z*. The angular emission characteristics demonstrate that horizontally-oriented dipole emitters generate strong forward-directional emission especially along the *x*-direction (*Px*). For organic materials that may be better modeled with randomly oriented dipoles, angular optical characterisitcs are now averaged with dipole emitters oriented along *x*, *y*, and *z* axes. Figure 3d shows the angular emission characteristics of MIB-OLED and planar B-OLED when light intensity is normalized by the maximum of MIB-OLED, i.e., ${I}_{MIB-OLED}\left(\theta \right)/{I}_{MIB-OLED}^{MAX}$ vs. ${I}_{B-OLED}\left(\theta \right)/{I}_{MIB-OLED}^{MAX}$. With an enhancement *E* defined as ${E\left(\theta \right)\stackrel{\scriptscriptstyle\mathrm{def}}{=}I}_{MIB-OLED}\left(\theta \right)/{I}_{B-OLED}\left(\theta \right)$, *E*($\theta$) exceeds unity for an angular range $\left|\theta \right|\le {46}{^\circ }$ with a maximum at 1.58-fold at an emission angle of 0°. As shown in Fig. 3e, the enhancement gradually decreases with an increase of the emission angle.

We can also define a viewing angle as the angle position where the intensity of MIB-OLED and B-OLED reaches the half of the respective maximum intensity. When compared with the standard Lambertian emission distribution as shown in Fig. 3f, MIB-OLED shows stronger forward directional emission with a viewing angle of 101° which is narrower than 122° of planar B-OLED. As a result, out-coupled emission of MIB-OLED much better approximates the emission from a Lambertian plane light source which is desired for light emitting source.

On the other hand, Figure 3g–l show spatial power distribution of MIB-OLED and planar B-OLED in the *zx* plane with respect to dipole orientations when time-averaged Poynting vectors represented by red arrows are placed on the top surface of a glass substrate. Despite equivalent internal radiation patterns that originate from Hertz dipole emitters, MIB-OLED shows narrower out-coupled angular emission characteristics than that of planar B-OLED, especially in cases of horizontally-aligned dipole emitters.

The results of Figs. 2, 3 suggest that MIB-OLED should exhibit stronger emission intensity while its angular emission distribution mimics that of a Lambertian source better than conventional planar B-OLED.

### Experimental confirmation

For experimental confirmation of the MIB-OLED model, we have fabricated a cavity structure integrated with MS and performed passive characterization of the structure. For a long time, many studies of nanostructure-integrated OLEDs have been conducted, where highly enhanced OCE of OLEDs has been experimentally validated^70–73^. In the case of ambient light reflection, however, high-contrast OLEDs have been proposed largely in conjunction with thin-film structures such as anti-reflection coating or black cathodes without any nanostructures^17–24^. For this reason, we have focused on experimental confirmation of reflection characteristics of ambient light to MS-integrated cavity structure. Organic materials of MIB-OLED such as Alq_3_, PEDOT:PSS, and TAPC are replaced with inorganic materials which have similar refractive indices for efficient and stable fabrication. Although passive characterization of the structure without complex fabrication processes does not provide a complete picture, it can still confirm the enhanced performance due to MS in the cavity.

**Figure 4:**
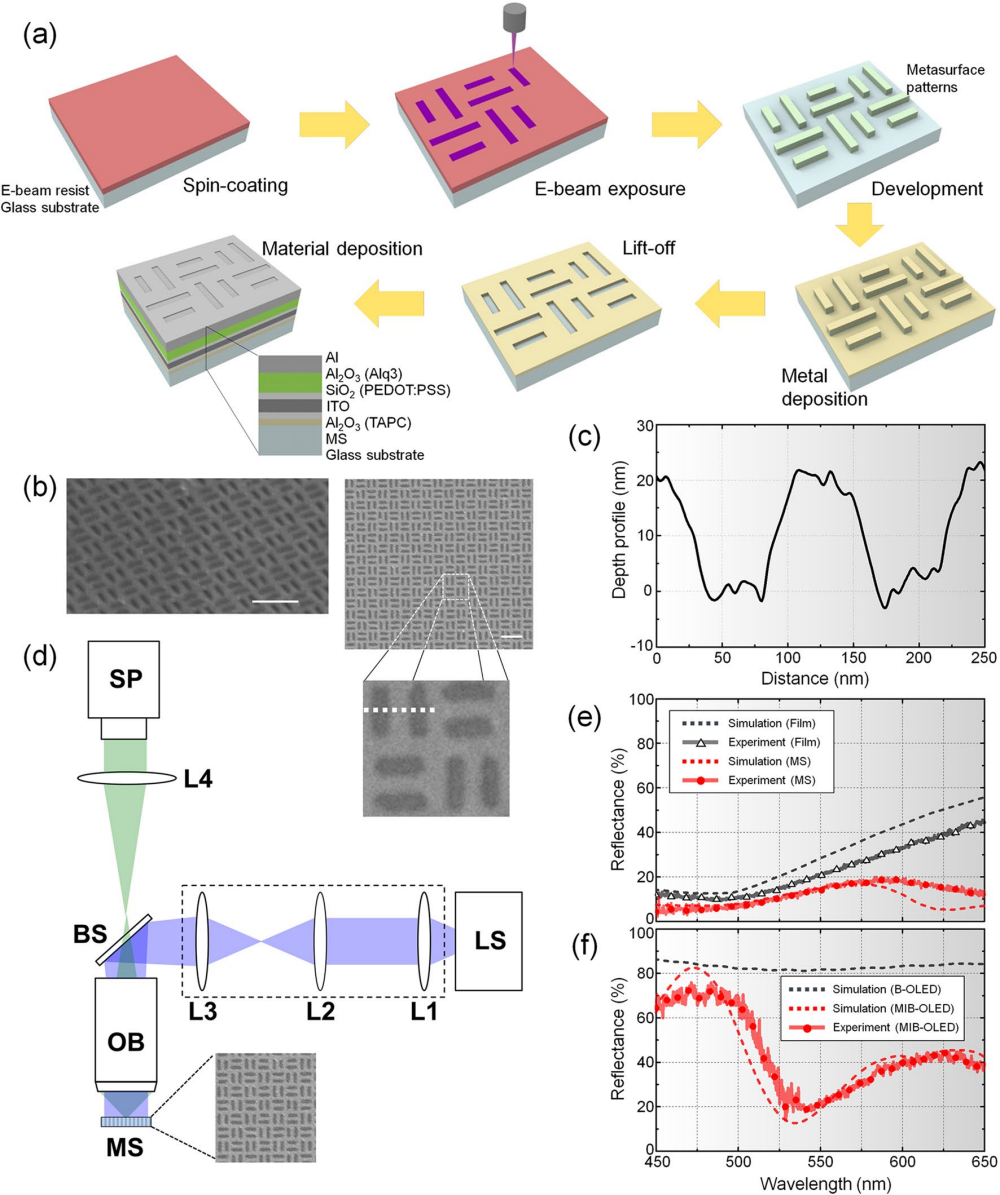
(**a**) Schematic diagram of the fabrication processes to implement the MIB-OLED model along with a final cross-section. (**b**) SEM image of a fabricated MS sample prior to material deposition (scale bars: 500 nm): tilted (left) and top view (middle). The magnified image on the right represents a unit MS pattern with a 500-nm size. (**c**) Depth profile of the measured SEM image of slots across the white dashed line in (**b**). (**d**) Schematic set-up for passive experimental characterization of MS for the MIB-OLED model (LS: light source, OB: objective lens, BS: beam splitter, SP: spectrometer, and L1–L4: collimation optics). (**e**) Measured and simulated reflectance spectra for only MS and gold thin film deposited on BK7 substrate. Thickness of MS and gold film is 20 nm and MS composed of mixed-slot arrays whose structural parameters of *L*_*1*_, *L*_*2*_, and *W* are fixed to 200, 160, and 60 nm, respectively. (**f**) Comparison of reflectance spectrum between conventional B-OLED and MS-integrated cavity structure. Integrated MS has a 20-nm thickness and its structural parameters of *L*_*1*_, *L*_*2*_, and *W* are 200, 160, and 60 nm.

The fabrication was largely based on electron beam lithography to define the nanoscopic size of the MS structures. The schematic illustration for the fabrication procedure is presented in Fig. 4a. MS used for experimental confirmation started with sonication of a BK7 glass substrate with acetone, IPA, and water for 5 minutes. A combined layer of adhesion promoter (AR 300-80) and E-beam resist (AR-N 7520.073) was spin-coated for 1 minute at 4000 rpm. A conductive protective layer (AR-PC 5091.02) was then spin-coated with 3000 rpm. In the patterning process, a periodic slot array was exposed with an electron beam in an area of 200 μm × 200 μm. This is followed by thermal deposition of a 20-nm thick Au thin film and a 2-nm Cr adhesion layer. The sample was immersed in the remover (AR 300-70) for 1 hour on a 150 °C hot plate to lift off the sacrificing resist pattern. The fabrication steps are illustrated in Fig. 4a. The final length of the experimentally fabricated two slots were *L*_*1*_ = 200 nm, *L*_*2*_ = 160 nm and *W* = 60 nm, as shown in Fig. 4b. After fabrication of the MS layer in gold, other materials were sequentially deposited for the MIB-OLED. The CPL and EML were replaced by Al_2_O_3_, and HIL by SiO_2_. SEM images of a fabricated MS sample are shown in Fig. 4b. More details of the depth profile can be provided by image analysis with improved clarity, i.e., after gray tones corresponding to the zero height were found, measured gray values can be converted into the depth scale, as shown in Fig. 4c, which confirms that the slots are indeed 20 nm thick.

To confirm large absorption achieved with MS, an optical set-up was constructed as shown in Fig. 4d. The set-up consists of a white-light lamp (FL-1039 Xenon arc lamp, Horiba, Japan) as light source and a spectrometer (HR4000, Ocean Optics, U.S.A.) with collimation optics. L1, L2, and L3 describe a collimating light illuminator (BX-RLA2, Olympus, Japan). L4 is a tube lens with *f* = 100 mm. An objective lens (UMPlanFl 100X NA0.9, Olympus) was used to illuminate and acquire reflected light from a MS sample. Firstly, reflectance spectra of MS were compared to that of gold thin film, both of which were formed on the BK7 substrate. The thickness of MS and gold thin film was fixed at 20 nm while structural parameters of mixed-slot arrays for the fabrication were *L*_*1*_ = 200 nm, *L*_*2*_ = 160 nm, and *W* = 60 nm. Incident light was unpolarized and normal to the MS and the gold thin film. Figure 4e shows an experimental reflectance spectrum of a MS sample (red solid line with symbols of filled circles) which is 1.9-fold lower on average than the reflectance of gold thin film (black solid line with symbols of open triangles) in the 450 to 650 nm visible wavelength range. These results are overall in good agreement with the calculated reflectance spectra, as shown in Fig. 4e (dashed lines in red and black). The discrepancy between experimental and numerical data appears to arise from the imperfection in the fabricated slot array size and shape of MS, which may have caused a shift in the wavelength at which LSP is induced. Nonetheless, the result confirms that MS produces large absorption of light, causing the excitation of LSP, which shows up as reduced reflectance.

The reflection of ambient light by the MS-integrated cavity structure was also measured in an identical set-up. Figure 4f shows the measured and simulated reflectance spectra in the 450 to 650 nm visible wavelength range. Measured results (red solid line with circular symbols) agree well with the simulation (dashed line in red). We have also compared the reflectance spectrum between conventional B-OLED (dashed in black) and MS-integrated cavity structure (dashed in red). Note that conventional B-OLED shows strong reflection (above 80%) due to the aluminum cathode in the whole visible wavelength range. By comparison, the MS-integrated cavity structure presents significantly lower reflection with high possibility to reduce luminous reflectance, especially in the range of 525 to 575 nm wavelength where eye sensitivity can be strongly associated. The reduced reflectance in the wavelength range 500–575 nm is associated with absorption by excited SPPs that arise from the coupling between Al electrode and gold nanoslot layer. The reflectance in the longer waveband may also be affected due to absorption by LSP excitation. It was found that the effect of SPPs is less affected by the nanoslot width than that of LSPs, i.e., the absorption peaks due to LSPs tend to be blue-shifted as the nanoslots become wider. The minimum reflectance was obtained as 13.8% at 529 nm (experiment) and 12.5% at 535 nm (simulation). This represents reduction of reflectance by about 5.9 and 6.5 times, respectively, in reference to 81.7% at 530 nm for conventional B-OLED. These results suggest that MIB-OLED can improve the optical performance significantly.

We now discuss changes in the electrical properties of OLEDs caused by the introduction of a MS layer. In the MIB-OLED structure shown in Fig. 1b, because MS is separated from anode by the capping layer (TAPC) and is not in direct contact with anode, electron-hole pair recombination is affected little by the MS and its effect on electric properties such as hole carrier mobility is weak. In other words, LSP absorption that is associated with reduced back reflection due to the MS tends not to influence carrier mobility significantly, though this may increase injected current density. Such as trend is overall confirmed in other studies, e.g., when SP localized by aluminum nanoparticles embedded in a HIL, hole carrier injection and transport were found not to be affected by LSP absorption^74^. Also, embedding gold nanoparticles in a HIL, while enhancing optical efficiency, did not significantly affect hole injection efficiency^75^. On the other hand, anode replaced with a metallic mesh electrode was found to decrease hole injection barrier height and thereby obtain high current density^76^. Furthermore, luminance efficiency is improved due to SP localization, for example, as a result of energy transfer between LSP and photon radiated in an EML or reduced lifetime of exciton through Purcell effect.

## Conclusion

In summary, we have investigated performance enhancement of B-OLEDs integrated with MS. Effect of layer thicknesses and structural parameters has been investigated to improve performance measures such as OCE and reflectance of the ambient light. Quantitative evaluation of performance was measured by an optimization factor *γ*. To the best of our knowledge, this is the first study to explore integration of MS to high-contrast OLED structure.

Improvement was first made by optimizing *d*_*CPL*_ and *d*_*MS*_: with optimization of layer thicknesses, performance of MIB-OLED was shown to enhance by merely 3.9% over conventional B-OLED with *γ*_*max*_ = 14.35. Further improvement was made by optimizing structural parameters *L*_*2*_ and *W* of MS. The maximum optimization factor was achieved as *γ*_*max*_ = 16.02, i.e. the enhancement associated with MS reaches 16%, compared to the conventional B-OLED. These structural parameters produce an OCE of 15.6% and reflectance at 26.9% and the OCE shows enhancement by 51% over that of B-OLED. Angular emission distribution of optimized MIB-OLED has a more Lambertian-like pattern than that of planar B-OLED. Enhanced optical performance of MIB-OLED is validated by experimental results. With higher *γ* to be achieved using more efficient optimization algorithms, we expect MS to be extremely useful as an element to improve the performance of conventional OLED structures.

## References

[1] Bulović V (1998). Weak microcavity effects in organic light-emitting devices. Phys. Rev. B.

[2] Gu G, Burrows PE, Venkatesh S, Forrest SR, Thompson ME (1997). Vacuum-deposited, nonpolymeric flexible organic light-emitting devices. Opt. Lett..

[3] Chang C-H (2010). Enhancing color gamut of white OLED displays by using microcavity green pixels. Org. Electron..

[4] Chu XB (2014). Fast responsive and highly efficient optical upconverter based on phosphorescent OLED. ACS Appl. Mater. Inter..

[5] Kim BC (2014). Wideband antireflective circular polarizer exhibiting a perfect dark state in organic light-emitting-diode display. Opt. Express.

[6] Tokito S, Tsutsui T, Taga Y (1999). Microcavity organic light-emitting diodes for strongly directed pure red, green, and blue emissions. J. Appl. Phys..

[7] Lin C-L, Lin H-W, Wu C-C (2005). Examining microcavity organic light-emitting devices having two metal mirrors. Appl. Phys. Lett..

[8] Park MJ (2014). High efficiency red top-emitting micro-cavity organic light emitting diodes. Opt. Express.

[9] Poitras D, Kuo CC, Py C (2008). Design of high-contrast OLEDs with microcavity effect. Opt. Express.

[10] Cok, R. S. OLED display with circular polarizer. U.S. Patent 7,259,505 (2007).

[11] Vaenkatesan V (2005). Improving the brightness and daylight contrast of organic light-emitting diodes. Adv. Funct. Mater..

[12] Xie WF, Zhao Y, Li CN, Liu SY (2006). Contrast and efficiency enhancement in organic light-emitting devices utilizing high absorption and high charge mobility organic layers. Opt. Express.

[13] Li J-F, Su S-H, Hwang K-S, Yokoyama M (2007). Enhancing the contrast and power efficiency of organic light-emitting diodes using CuPc/TiOPc as an anti-reflection layer. J. Phys. D Appl. Phys..

[14] Lau KC, Xie WF, Sun HY, Lee CS, Lee ST (2006). Contrast improvement of organic light-emitting devices with Sm: Ag cathode. Appl. Phys. Lett..

[15] Xie WF (2006). High-contrast and high-efficiency top-emitting organic light-emitting devices. Appl. Phys..

[16] Chen SM, Yuan YB, Lian JR, Zhou X (2007). High-efficiency and high-contrast phosphorescent top-emitting organic light-emitting devices with p-type Si anodes. Opt. Express.

[17] Kim YH (2011). High contrast green OLEDs using inorganic metal multi layer. Synth. Methods.

[18] Zhou YC (2006). High contrast organic light-emitting devices with improved electrical characteristics. Appl. Phys. Lett..

[19] Chiu T-L (2011). Absorptive and conductive cavity cathode with silver nanoparticles for low-reflection organic light-emitting devices. J. Phys. D Appl. Phys..

[20] Man J-X (2017). Black phase-changing cathodes for high-contrast organic light-emitting diodes. ACS Photonics.

[21] Chen S, Xie J, Yang Y, Chen C, Huang W (2010). High-contrast top-emitting organic light-emitting diodes with a Ni/ZnS/CuPc/Ni contrast-enhancing stack and a ZnS anti-reflection layer. J. Phys. D Appl. Phys..

[22] Yang C-J (2005). High-contrast top-emitting organic light-emitting devices for active-matrix displays. Appl. Phys. Lett..

[23] Lee BD (2008). Characteristics of contrast of active-matrix organic light-emitting diodes containing a black matrix and antireflection layers. Mater. Chem. Phys..

[24] Tan G (2016). High ambient contrast ratio OLED and QLED without a circular polarizer. J. Phys. D Appl. Phys..

[25] Cho H, Jin C, Kim E, Yoo S (2014). Polarizer-free, high-contrast-ratio organic light-emitting diodes utilizing microcavity structures and neutral-density filters. J. Inf. Disp..

[26] Kim S-Y, Lee J-H, Lee J-H, Kim J-J (2012). High contrast flexible organic light emitting diodes under ambient light without sacrificing luminous efficiency. Org. Electron..

[27] Ding B-F, Alameh K (2012). High-contrast tandem organic light-emitting devices employing semitransparent intermediate layers of LiF/Al/C_60_. J. Phys. Chem. C.

[28] Yang C-J, Cho T-Y, Lin C-L, Wu C-C (2008). Energy-recycling high-contrast organic light-emitting devices. J. Soc. Inf. Disp..

[29] Adato R (2009). Ultra-sensitive vibrational spectroscopy of protein monolayers with plasmonic nanoantenna arrays. Proc. Natl. Acad. Sci..

[30] Kim K (2017). Molecular overlap with optical near-fields based on plasmonic nanolithography for ultrasensitive label-free detection by light-matter colocalization. Biosens. Bioelectron..

[31] Liu N, Mesch M, Weiss T, Hentschel M, Giessen H (2010). Infrared perfect absorber and its application as plasmonic sensor. Nano Lett..

[32] Lee C, Sim E, Kim D (2017). Effect of nanogap-based light-matter colocalization on the surface plasmon resonance detection. J. Light. Technol..

[33] Atwater HA, Polman A (2010). Plasmonics for improved photovoltaic devices. Nat. Mater..

[34] Kawata S, Inouye Y, Verma P (2009). Plasmonics for near-field nano-imaging and superlensing. Nat. Photonics.

[35] Lee W (2015). Three-dimensional super localization imaging of gliding Mycoplasma mobile by extraordinary light transmission through arrayed nanoholes. ACS Nano.

[36] Son T (2019). Superlocalized three-dimensional live imaging of mitochondrial dynamics in neurons using plasmonic nanohole arrays. ACS Nano.

[37] Lee H (2020). Surface plasmon localization-based super-resolved Raman microscopy. Nano Lett..

[38] Xu T, Wu YK, Luo XG, Guo LJ (2010). Plasmonic nanoresonators for high-resolution colour filtering and spectral imaging. Nat. Commun..

[39] Wu Y-KR, Hollowell AE, Zhang C, Guo LJ (2013). Angle-insensitive structural colours based on metallic nanocavities and coloured pixels beyond the diffraction Limit. Sci. Rep..

[40] Zeng BB, Gao YK, Bartoli FJ (2013). Ultrathin nanostructured metals for highly transmissive plasmonic subtractive color filters. Sci. Rep..

[41] Rajasekharan R (2014). Filling schemes at submicron scale: Development of submicron sized plasmonic colour filters. Sci. Rep..

[42] Akselrod GM (2015). Large-area metasurface perfect absorbers from visible to near-infrared. Adv. Mater..

[43] Cheng F, Gao J, Luk TS, Yang XD (2015). Structural color printing based on plasmonic metasurfaces of perfect light absorption. Sci. Rep..

[44] Hedayati MK (2011). Design of a perfect black absorber at visible frequencies using plasmonic metamaterials. Adv. Mater..

[45] Cao T, Wei C-W, Simpson RE, Zhang L, Cryan MJ (2014). Broadband polarization-independent perfect absorber using a phase-change metamaterial at visible frequencies. Sci. Rep..

[46] Duan X (2014). Polarization-insensitive and wide-angle broadband nearly perfect absorber by tunable planar metamaterials in the visible regime. J. Opt..

[47] Zhou L (2016). Tailoring directive gain for high-contrast, wide-viewing-angle organic light-emitting diodes using speckle image holograpy metasurfaces. ACS Appl. Mater. Intererfaces.

[48] Joo W-J (2020). Metasurface-driven OLED displays beyond 10,000 pixels per inch. Science.

[49] Huh JW (2012). The optical effects of capping layers on the performance of transparent organic light-emitting diodes. IEEE Photonics J..

[50] Lee W-K (2018). Quantitative analyses of high electroluminescence efficiency of thermally activated delayed fluorescence emitters based on acridine-triazine hybrids. J. Photon. Energy.

[51] Hobson PA, Wasey JAE, Sage I, Barnes WL (2002). The role of surface plasmons in organic light-emitting diodes. IEEE J. Sel. Top. Quantum Electron..

[52] Kang K, Lee Y, Kim J, Lee H, Yang B (2016). A generalized Fabry-Pérot formulation for optical modeling of organic light-emitting diodes considering the dipole orientation and light polarization. IEEE Photonics J..

[53] Miyamaru F, Takeda MW (2009). Coupling between localized resonance and excitation of surface waves in metal hole arrays. Phys. Rev. B.

[54] Schwind M, Kasemo B, Zorić I (2013). Localized and propagating plasmons in metal films with nanoholes. Nano Lett..

[55] Kang K, Yoon J, Kim J, Lee H, Yang B (2015). Effect of the finite pixel boundary on the angular emission characteristics of top-emitting organic light-emitting diodes. Opt. Express.

[56] Park W-Y, Cheong H-W, Lee C, Whang K (2016). Design of highly efficient RGB top-emitting organic light-emitting diodes using finite element method simulations. Opt. Express.

[57] Kang K, Kim J (2019). Effect of dipole orientation on the angular emission characteristic of a three-dimensional top-emitting organic light-emitting diode with square pixel boundary. J. Korean Phys. Soc..

[58] Wang ZB (2011). Optical design of organic light emitting diodes. J. Appl. Phys..

[59] Im S, Sim E, Kim D (2018). Microscale heat transfer and thermal extinction of a wire-grid polarizer. Sci. Rep..

[60] Ohmori Y (2004). Realization of polymeric optical integrated devices utilizing organic light-emitting diodes and photodetectors fabricated on a polymeric waveguide. IEEE J. Sel. Top. Quantum Electron..

[61] Oka N (2010). Thermal diffusivities of tris (8-hydroxyquinoline) aluminum and N,Nʹ-di (1-naphthyl)-N,Nʹ-diphenylbenzidine thin films with sub-hundred nanometer thicknesses. Jpn. J. Appl. Phys..

[62] Bergemann KJ, Krasny R, Forrest SR (2012). Thermal properties of organic light-emitting diodes. Org. Electron..

[63] Lee CJ, Pode RB, Han JI, Moon DG (2007). Ca/Ag bilayer cathode for transparent white organic light-emitting devices. Appl. Surf. Sci..

[64] Kang K (2016). A simple numerical modeling of the effect of the incoherent thick substrate in thin-film solar cells based on the equispaced thickness method. IEEE Photonics J..

[65] Kang K, Kim S, Kim J (2018). Numerical modeling of the effect of multiple incoherent layers in Cu(In, Ga)S_e_2 solar cells based on the equispaced thickness averaging method. Appl. Opt..

[66] Benisty H, Neve HD, Weisbuch C (1998). Impact of planar microcavity effects on light extraction—Part I: Basic concepts and analytical trends. IEEE J. Sel. Top. Quantum Electron..

[67] Hobson PA, Wedge S, Wasey JAE, Sage I, Barnes WL (2002). Surface plasmon mediated emission from organic light-emitting diodes. Adv. Mater..

[68] Fujiki A (2010). Enhanced fluorescence by surface plasmon coupling of Au nanoparticles in an organic electroluminescence diode. Appl. Phys. Lett..

[69] Lee H, Kim D (2016). Curvature effects on flexible surface plasmon resonance biosensing: Segmented-wave analysis. Opt. Express.

[70] Do YR, Kim Y-C, Song Y-W, Lee Y-H (2004). Enhanced light extraction efficiency from organic light emitting diodes by insertion of a two-dimensional photonic crystal structure. J. Appl. Phys..

[71] Shin J-W (2014). Random nano-structures as light extraction functionals for organic light-emitting diode applications. Org. Electron..

[72] Lim T-B, Cho KH, Kim Y-H, Jeong Y-C (2016). Enhanced light extraction efficiency of OLEDs with quasiperiodic diffraction grating layer. Opt. Express.

[73] Hwang H (2018). Nano-arrayed OLEDs: Enhanced outcoupling efficiency and suppressed efficiency roll-off. Nanoscale.

[74] Khadir S, Diallo A, Chakaroun M, Boudrioua A (2017). Exciton enhancement and exciplex quenching by plasmonic effect of Aluminum nanoparticle arrays in a blue organic light emitting diode. Opt. Express.

[75] Xiao Y (2012). Surface plasmon-enhanced electroluminescence in organic light-emitting diodes incorporating Au nanoparticles. Appl. Phys. Lett..

[76] Ding W, Wang YX, Chen H, Chou SY (2014). Plasmonic nanocavity organic light-emitting diode with significantly enhanced light extraction, contrast, viewing angle, brightness, and low-glare. Adv. Funct. Mater..

